# Comparison between Dexamethasone and Methylprednisolone Therapy in Patients with COVID-19 Pneumonia Admitted to Non-Intensive Medical Units

**DOI:** 10.3390/jcm10245812

**Published:** 2021-12-12

**Authors:** Roberta Buso, Francesco Cinetto, Alessandro Dell’Edera, Nicola Veneran, Cesarina Facchini, Valeria Biscaro, Stefania Schiavon, Elisa Vian, Ugo Grossi, Giacomo Zanus, Mario Giobbia, Riccardo Scarpa, Carlo Agostini, Marcello Rattazzi, Carla Felice

**Affiliations:** 1 Medicine 1 Unit, Department of Medicine, Ca’ Foncello University Hospital, 31100 Treviso, Italy; roberta.buso@aulss2.veneto.it (R.B.); francesco.cinetto@unipd.it (F.C.); alessandro.delledera@aulss2.veneto.it (A.D.); nicola.veneran@studenti.unipd.it (N.V.); cesarina.facchini@aulss2.veneto.it (C.F.); riccardo.scarpa@aulss2.veneto.it (R.S.); carlo.agostini@unipd.it (C.A.); carla.felice@aulss2.veneto.it (C.F.); 2 Department of Medicine, DIMED, University of Padova, 35122 Padova, Italy; 3 Microbiology Unit, Department of Specialist and Laboratory Medicine, Ca’ Foncello University Hospital, 31100 Treviso, Italy; valeria.biscaro@aulss2.veneto.it (V.B.); stefania.schiavon@aulss2.veneto.it (S.S.); elisa.vian@aulss2.veneto.it (E.V.); 4 Surgery 2 Unit, DISCOG, Ca’ Foncello University Hospital, University of Padua, 31100 Treviso, Italy; ugo.grossi@aulss2.veneto.it (U.G.); giacomo.zanus@aulss2.veneto.it (G.Z.); 5 Infective Disease Unit, Department of Specialist and Laboratory Medicine, Ca’ Foncello University Hospital, 31100 Treviso, Italy; mario.giobbia@aulss2.veneto.it

**Keywords:** SARS-CoV-2, COVID-19, steroid therapy, methylprednisolone, dexamethasone

## Abstract

(1) Background: Data on different steroid compounds for the treatment of hospitalized COVID-19 (coronavirus disease 2019) patients are still limited. The aim of this study was to compare COVID-19 patients admitted to non-intensive units and treated with methylprednisolone or dexamethasone. (2) Methods: This was a single-center retrospective study that included consecutive patients with COVID-19 hospitalized in medical wards during the second wave of the pandemic. Thirty-day mortality and the need for intensive or semi-intensive care were the main clinical outcomes analyzed in patients receiving methylprednisolone (60 mg/day) compared with dexamethasone (6 mg/day). Secondary outcomes included complication rates, length of hospital stay, and time to viral clearance. (3) Results: Two-hundred-forty-six patients were included in the analysis, 110 treated with dexamethasone and 136 with methylprednisolone. No statistically significant differences were found between the two groups of patients regarding 30-day mortality (OR 1.35, CI95% 0.71–2.56, *p* = 0.351) and the need for intensive or semi-intensive care (OR 1.94, CI95% 0.81–4.66, *p* = 0.136). The complication rates, length of hospital stay, and time to viral clearance did not significantly differ between the two groups. (4) Conclusions: In patients hospitalized for COVID-19 in non-intensive units, the choice of different steroid compounds, such as dexamethasone or methylprednisolone, did not affect the main clinical outcomes.

## 1. Introduction

The spread of the novel SARS-CoV-2 (severe acute respiratory syndrome coronavirus 2), responsible for the disease known as COVID-19 (coronavirus disease-2019), has led to the outbreak of a global pandemic, which has so far caused more than 4.5 million confirmed deaths [1]. The majority of SARS-CoV-2 infections are asymptomatic or result only in mild disease [2]. However, some patients develop interstitial bilateral pneumonia, which can progress to critical illness with respiratory failure and death [3,4,5]. The main pathophysiological features of severe COVID-19 are driven by an acute inflammatory response that promotes diffuse alveolar damage, inflammatory infiltrates, and microvascular thrombosis [6,7,8].

The lack of therapeutic options was one of the most significant issues when facing the initial phase of the COVID-19 pandemic, both in symptomatic and asymptomatic patients [9,10,11,12]. Several therapeutic strategies were proposed and tested in randomized controlled trials [13,14,15,16]. Among these, the RECOVERY study showed that the administration of dexamethasone (DEX) in COVID-19 patients with severe pulmonary involvement reduced the risk of mortality compared to usual care [17]. As a consequence, the administration of steroids in patients with COVID-19 pneumonia is now recognized as an effective therapeutic option and it is recommended for the management of SARS-CoV-2 infection. A recent meta-analysis confirmed the efficacy of steroid therapy in the management of patients with COVID-19 pneumonia, but we still lack a definitive indication about a gold standard molecule or specific dosage [18]. In particular, it is still unclear whether the use of other glucocorticoids can exert clinical protection similar to DEX. For instance, only a few studies investigated the efficacy of methylprednisolone (MP) versus placebo against COVID-19 pneumonia [19,20,21], with inconclusive results or early stoppage because of the already available data from the RECOVERY study. 

Currently, there are no real-life data regarding the use of different steroid products for the treatment of hospitalized COVID-19 patients. Furthermore, most of the available studies included patients with severe COVID-19 that were already treated in intensive care units (ICUs), whereas data on early treatment with steroids in order to prevent patient deterioration and transfer to ICU are still lacking. Therefore, we performed a retrospective analysis of the effects of two different glucocorticoid regimens (MP versus DEX) on clinical outcomes in patients with COVID-19 pneumonia that were specifically admitted to non-intensive medical wards.

## 2. Materials and Methods

### 2.1. Study Population

This was a single-center retrospective study that included consecutive adult patients with COVID-19 pneumonia that were admitted to non-intensive units at the Regional Hospital of Treviso (Italy) between the 1st December 2020 and the 31st January 2021. Each diagnosis of SARS-CoV-2 infection was made using semi-quantitative real-time reverse transcription-polymerase chain reaction (RT-PCR) on a nasopharyngeal swab, according to international guidelines. 

Inclusion criteria were admission to medical wards directly from the Emergency Department, the presence of pneumonia detected using imaging (chest X-ray or pulmonary computed tomography (CT)), and the need for oxygen supplementation. Criteria for hospital admission of COVID-19 patients were established by local protocols and remained unchanged throughout the observation period. 

For the present analysis, we considered all the patients treated using dexamethasone 6 mg once daily (DEX) or methylprednisolone 60 mg/day (MP), both given intravenously. The treatment choice for each patient was arbitrarily determined by the clinician. The dose of MP could be given either once daily or in two fractionated doses. The treatment was started at the time of admission and continued for at least 10 days; then, the dose was gradually tapered. The tapering process was not established by a specific protocol; therefore, the overall duration of the steroid therapy may have possibly varied between patients, depending on the tolerance and medical indications after discharge. Patients transferred from other hospital units, treated with different steroid doses, or with no need for oxygen therapy were excluded.

Patients’ demographic and clinical characteristics were retrieved from medical records and entered into an anonymous database. Data included age; gender; active smoking; medical history, including the presence and the type of comorbidities, such as cardiovascular diseases (myocardial infarction/stroke/heart failure, atrial fibrillation), obesity (BMI ≥30 kg/m^2^), dyslipidemia, hypertension, chronic obstructive pulmonary disease (COPD), diabetes, chronic kidney disease (CKD), and cancer (previous or active); and all concomitant chronic medical treatments. Moreover, the type and duration of COVID-19-related symptoms, clinical parameters and the SOFA score at admission, blood tests results, any specific additional therapy during hospitalization (such as antibiotic, Remdesivir, anticoagulant or antiplatelet drugs, and convalescent plasma), length of stay, and COVID-19-related outcomes (need for semi-intensive or intensive unit care and death) were recorded. 

The study was approved by the local Research Ethics Committee (793/CE Marca Trevigiana).

### 2.2. Primary and Secondary Outcomes 

The primary outcomes of our study were all-cause mortality within 30 days from the hospital admission and the need for semi-intensive care unit (s-ICU) or ICU admission for non-invasive (NIV) or mechanical ventilation.

The secondary endpoints of our study included the length of hospital stay (time from hospital admission to discharge); time of viral clearance (time from the diagnosis of infection to the first negative RT-PCR swab); rate and type of complications, such as thromboembolic events, hemorrhage, cardiovascular disease (myocardial infarction, stroke, heart failure, new diagnosis of atrial fibrillation), acute renal injury, other infections, and a new diagnosis of diabetes.

### 2.3. Statistical Analysis

Continuous variables were reported as mean and standard deviation (SD), whereas categorical variables were reported as percentages. Baseline data comparisons between groups of continuous variables were performed by using Student’s t-test, whereas the chi-square test was used for the comparison of categorical variables. A binary logistic regression analysis was performed to estimate the univariate and multivariate odds ratios (OR) and 95% confidence intervals (CI) for the risk of 30-day all-cause mortality and the need for s-ICU/ICU admission (treatment with DEX was considered as a reference). Multivariable logistic regressions were performed, taking into consideration the following covariates: age, gender, comorbidities, use of antibiotics, use of Remdesivir, and duration of symptoms before hospital admission. Kaplan–Meier survival analysis was also performed to investigate the 30-day and s-ICU/ICU-admission-free survival. The log-rank (Mantel–Cox) test was used for the comparison between the survival curves of the two groups. Statistical significance was considered as a two-tailed *p* < 0.05. All the analyses were performed using IBM SPSS statistics 27.0.

## 3. Results

### 3.1. Study Population

In the period under investigation, a total of 442 patients were admitted to the Medical Department with a diagnosis of COVID-19 pneumonia. After admission, 110 patients were treated with DEX and 136 with MP. Eight patients did not receive steroids treatment, whereas 69 subjects were treated with different compounds or doses (i.e., MP < 60 mg/day, oral prednisone, etc.). 

The baseline characteristics of the 246 patients included in the study are summarized in Table 1. In particular, we observed that subjects treated with DEX or MP had similar mean ages and there was an equal gender distribution, with a higher prevalence of males in both groups (67.5% of the whole population). The two groups of patients also displayed a similar prevalence of comorbidities and an identical background of ongoing medications. The most prevalent diseases were hypertension, dyslipidemia, and diabetes, which were equally distributed in the two populations. We observed a higher prevalence of chronic kidney disease (CKD) and cancer only in the DEX group. 

Patients treated with DEX or MP also showed similar rates and types of COVID-19-related symptoms (Table S1), as well as identical clinical presentation and biochemical data at admission (Table 2 and Table S2). In particular, DEX and MP patients had similar mean values SOFA scores and PaO_2_/FiO_2_ ratios, whereas biochemical investigations showed a comparable rise in the two groups of C-reactive protein (CRP), ferritin, and D-dimer levels. 

During the hospital stay, most of the patients included in the analysis were treated with anticoagulant agents as either a prophylactic or at full dose. The majority of patients also received antibiotics (71.1%), mostly cephalosporin, followed by tetracyclines and azithromycin. The administration of antibacterial drugs was significantly higher in the MP group (MP 79.4% versus DEX 60.9%, *p* = 0.001) and the antiviral treatment with Remdesivir was used in a higher percentage of patients treated with MP (MP 54.4% versus DEX 41.8%, *p* = 0.049, Table 3).

### 3.2. Clinical Outcomes

**Figure 1:**
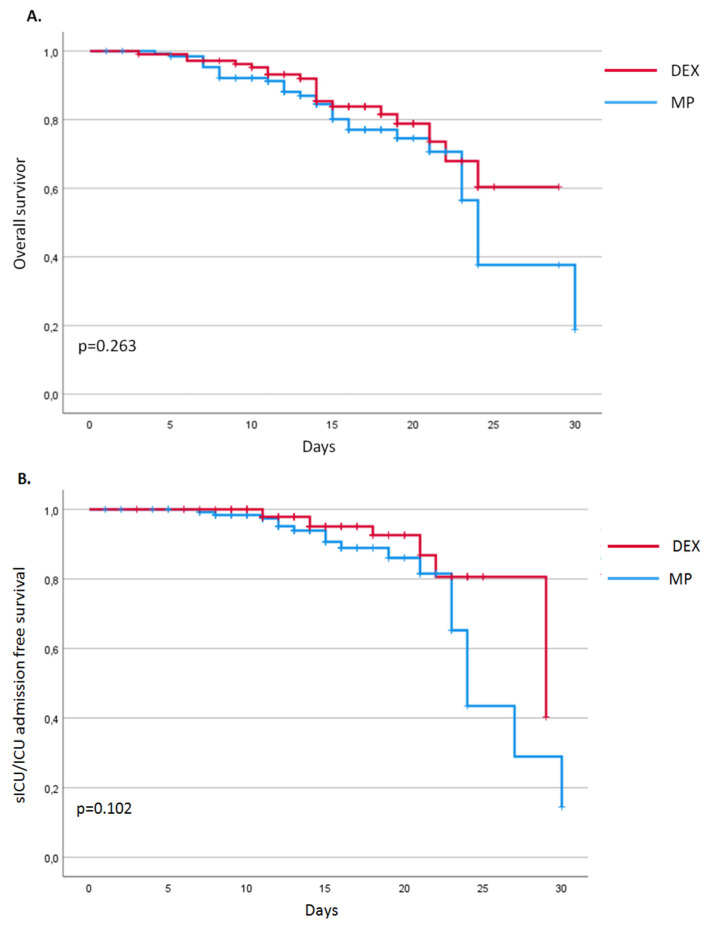
Overall 30-days (**A**) and sICU/ICU-admission-free (**B**) survival.

The overall 30-day mortality observed in our study was 19.9% (49 patients), without evidence of a different distribution between patients treated with DEX or MP. In particular, we observed 19 deaths in the DEX group (17.3%) and 30 deaths in patients treated with MP (22.1%) (*p* = 0.350). A total of 26 subjects (10.6%) were admitted to sICU/ICU, with a similar distribution between the two groups of patients (DEX: 8 patients, 7.3%; MP: 18 patients, 13.2%; *p* = 0.310). As a consequence, we did not observe a significant difference in 30-day mortality or s-ICU/ICU admission between the two treatment regimens (respectively OR 1.35, CI95% 0.71–2.56, *p* = 0.351 and OR 1.94, CI 95% 0.81–4.66, *p* = 0.136) (Table 4). This result was confirmed for both mortality (OR 0.78, CI 95% 0.35–1.72, *p* = 0.544) and risk of s-ICU/ICU admission (OR 1.37, CI 95% 0.54–3.46, *p* = 0.503) using a multivariate analysis adjusted for age, gender, comorbidities, use of antibiotics, treatment with Remdesivir, and symptom duration before admission (Table 4). The Kaplan–Meier survival analysis confirmed these findings, showing an overlap in the curves of DEX and MP for both 30-day overall and s-ICU/ICU-admission-free survival (Figure 1A).

Among patients with age ≥80 years who died because of COVID-19 pneumonia, some were not admitted to the ICU mainly due to their advanced age and comorbidities. To exclude a possible further interference of age on the main outcomes, a separate analysis was conducted in two subsets of patients (< or ≥80 years old) and the results confirmed that the steroid compound did not significantly affect the 30-day mortality and ICU admission risks (see Supplementary Tables S3 and S4).

**Figure 2:**
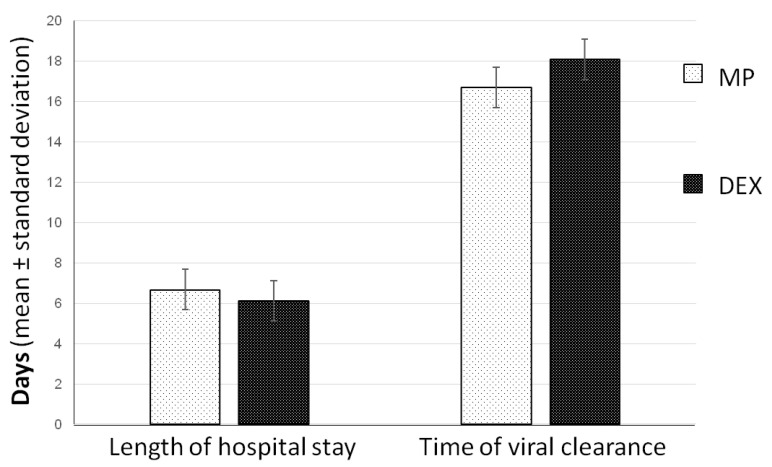
Comparison of mean length of hospital stay and time of viral clearance (expressed in days) between the two groups.

The temporal outcomes considered were the length of hospital stay and the time of viral clearance. Both these outcomes did not show any difference between the two groups (Figure 2). The mean time of hospital stay was 6.1 ± 0.4 days in the DEX group and 6.8 ± 0.5 days in the MP group (*p* = 0.390). The mean time of healing was 18.1 ± 0.5 days in the DEX group and 16.7 ± 0.6 days in the MP group (*p* = 0.940).

Complication rates during hospitalization were similar between the two groups (*p* = 0.278), with pulmonary embolism and infections representing the most frequent clinical events (Table S5). No significant differences were found in terms of secondary infections and diabetes between patients receiving the two steroid compounds (Table S5). 

## 4. Discussion

In this study, we compared two different steroid regimens (DEX or MP) adopted during the second wave of the pandemic to treat patients admitted to non-intensive medical wards because of COVID-19 pneumonia. No significant differences were found in the main clinical outcomes because the two groups of patients showed similar rates of s-ICU/ICU admission and 30-day mortality. Furthermore, the length of hospital stay, the time to viral clearance, and complication rates did not significantly differ between patients receiving DEX or MP. 

The natural course of SARS-CoV-2 infection may vary from asymptomatic to mild, moderate, or severe disease, with the development of pneumonia, acute respiratory distress syndrome, sepsis, multiorgan failure, and death [2,7]. After the initial acute phase of infection, most of the subsequent pulmonal and systemic tissue damage seems to be due to an imponent activation of the immune system, which causes a “cytokine storm” and directly damages the respiratory tract or the other organs [7,8]. Therefore, it was hypothesized that steroids may be an effective treatment for COVID-19 patients given their well-known anti-inflammatory and immunosuppressor effects. Several trials have been conducted to confirm this hypothesis [17,18,20,22]. The RECOVERY study is an open-label trial that includes a high number of patients treated with dexamethasone 6 mg once daily (n = 2104) or with standard care (n = 4321), showing that the steroid administration may reduce the 28-day mortality, especially in patients receiving oxygen support (with both non-invasive and invasive ventilation) [17]. Another trial (MetCOVID) compared the use of methylprednisolone 0.5 mg/kg twice daily for 5 days to placebo, showing a reduction in the 28-day mortality rate in patients older than 60 years, who had higher levels of C-reactive protein [20]. Moreover, recent meta-analyses seemed to confirm that steroids reduce mortality rates in hospitalized COVID-19 patients, with no increase in adverse events [18,23]. A small randomized trial compared MP 2 mg/kg/day and DEX 6 mg/day in 42 and 44 patients, respectively, showing better outcomes in the MP group [21]; however, the significantly higher dose of MP used in this study, considering the dose equivalence to DEX [24], and the small sample size make these results unreliable. Therefore, our study may add an important contribution to the specific clinical setting of the early treatment of patients with no severe COVID-19 at admission. We analyzed and compared COVID-19-related outcomes between patients that received two different steroid regimens, namely, DEX and MP, showing no significant differences in terms of 30-day mortality and s-ICU/ICU admission. With regard to the compound-specific pharmacokinetic, DEX seems to have stronger anti-inflammatory effects and longer action duration [24], whereas MP showed a high level of lung penetration [25]. These differences may potentially influence the clinical outcomes of patients with pneumonia; however, our findings indicate that patients hospitalized for SARS-CoV-2 pneumonia may benefit from steroid therapy regardless of the specific compound used. 

Our analysis was conducted in a specific clinical setting in that only patients admitted to a non-intensive medical unit, and therefore with moderate clinical activity of COVID-19 at admission, were enrolled. Therefore, these findings cannot be generalized and applied to all in-hospital settings. 

The correct timing of steroid administration during SARS-CoV-2 infection is still debated: if given too early, steroids may potentially prolong viral clearance and be ineffective due to the absence of inflammatory activation; on the other hand, if given too late, the inflammatory process that leads to tissue damage can be difficult to be reversed. In our study, steroids were administered after a mean of 7 +/− 3.5 days in the MP group and 5.5 +/− 3 days in the DEX group since the onset of SARS-CoV-2 infection, starting at the time of hospital admission, resulting in an earlier intervention compared to other reports. For example, in the MetCOVID study, patients started to receive MP or placebo after a median of 13 days since illness onset and the 28-day mortality rate was higher (37.7%) compared to other clinical records [20]. In the RECOVERY trial, the median time since the beginning of symptoms and the steroid treatment was shorter (median of 8 days) and closer to our data, and our overall mortality rate (19.9%) was similar to that reported in the group treated with DEX in the RECOVERY study (22.9%). [17] Rates of need for intensive care and death in our population were comparable to those reported in similar studies [17,23].

The main limitations of our study were the retrospective design and the absence of a control group not treated with steroids, which was impossible to be selected because, during the study period, only eight patients admitted to our hospital did not receive any type or dose of steroids. Furthermore, the overall duration of steroid therapy and BMI, which may potentially influence the considered outcomes, and the former smoking habit, were not recorded in our database. Our study population was naïve to the SARS-CoV-2 vaccine because, during the study period, only healthcare workers had access to the vaccine program in Italy; therefore, further data will be needed to confirm steroid utility for the treatment of hospitalized vaccinated patients. On the other hand, our study presents several strengths: First, compared to most observational studies or controlled trials published so far about steroids and COVID-19, the number of patients included in our analysis was high. Furthermore, baseline demographic and clinical characteristics of the two groups of patients were similar, making all comparisons robust and reliable. Moreover, local protocols for hospital admission and infection management remained unchanged throughout the entire study period; therefore, the study population can be considered homogeneous. 

In conclusion, our study offers novel insights into the therapeutic management of patients with COVID-19 admitted to non-intensive medical units. Increasing our knowledge about SARS-CoV-2 infection therapy is still crucial until the vaccines guarantee adequate coverage around the world. These real-world findings may contribute to reinforcing the results coming from larger controlled trials already published or still ongoing.

## Figures and Tables

**Table 1:** Baseline characteristics of the study population.

	Methylprednisolone (n = 136)	Dexamethasone (n = 110)	*p*
Age, years (mean, SD)	74.4 (13.2)	72.1 (12.1)	0.166
Gender—male, n (%)	92 (67.6)	74 (67.3)	0.950
Active smoker *, n (%)	5 (3.7)	2 (1.8)	0.383
Comorbidities, n (%)	119 (87.5)	101 (91.8)	0.273
Hypertension, n (%)	85 (62.5)	80 (72.7)	0.090
Obesity, n (%)	22 (16.2)	19 (17.3)	0.819
Diabetes, n (%)	29 (21.3)	29 (26.4)	0.354
Dyslipidemia, n (%)	39 (28.7)	24 (21.8)	0.220
Neoplasm, n (%)	23 (16.9)	33 (30.0)	0.015
CAD, n (%)	16 (11.8)	11 (10.0)	0.660
Atrial fibrillation, n (%)	16 (11.8)	11 (10.0)	0.660
VTE, n (%)	8 (5.9)	4 (3.6)	0.416
COPD, n (%)	9 (6.6)	4 (3.6)	0.299
CKD, n (%)	6 (4.4)	13 (11.8)	0.031
Concomitant chronic medications, n (%)	112 (82.4)	99 (90.0)	0.088
Systemic steroids, n (%)	6 (4.4)	5 (4.5)	0.960
Antihypertensive, n (%)	90 (66.2)	80 (72.7)	0.269
Antidiabetic, n (%)	25 (18.4)	26 (23.6)	0.312
Oral anticoagulant, n (%)	24 (17.6)	17 (15.5)	0.646
Antiplatelet, n (%)	38 (27.9)	31 (28.2)	0.967
Statins, n (%)	39 (28.7)	36 (32.7)	0.493
**Clinical Characteristics at Admission**			
Days from the onset to admission, mean (SD)	7.00 (3.5)	5.54 (3.8)	0.002
SOFA score, mean (SD)	3.85 (1.4)	4.20 (1.5)	0.330

* Only patients who currently smoke. Abbreviations: SD, standard deviation; CAD, coronary artery disease; VTE, venous thromboembolism; CKD, chronic kidney disease; COPD, chronic obstructive pulmonary disease.

**Table 2:** Main laboratory results at admission (all data are expressed as mean and standard deviation (SD)).

	Methylprednisolone (n = 136)	Dexamethasone (n = 110)	*p*
PaO_2_/FiO_2_ (mmHg)	250 (84)	261 (76)	0.298
PaO_2_ (mmHg)	68 (19)	69 (22)	0.770
WBC (10^3^/μL)	7.25 (3.82)	8.28 (10.85)	0.306
CRP (mg/dL)	8.91 (7.09)	8.34 (6.81)	0.529
Procalcitonin (ng/mL)	0.69 (3.2)	0.38 (0.57)	0.553
Lactate (mmol/L)	1.4 (0.6)	1.4 (0.6)	0.723
Ferritin (ng/mL)	970 (914)	961 (706)	0.946
Hemoglobin	134 (15)	134 (14)	0.865
Creatinin (mg/dL)	1.01 (0.43)	1.06 (0.62)	0.448
D-Dimer (ng/mL)	3234 (11261)	2530 (5808)	0.585
NT-ProBNP (pg/L)	1982 (5267)	1943 (4781)	0.955
T Troponin (ng/L)	32 (65)	31 (46)	0.941

Abbreviations: SD, standard deviation; WBC, white blood cell; CRP, C-reactive protein.

**Table 3:** Concomitant treatment during hospital stay.

Treatment	Methylprednisolone (n = 136)	Dexamethasone (n = 110)	*p*
Antibiotics, n (%)	108 (79.4)	67 (60.9)	0.001
Anticoagulant prophylaxis, n (%)	107 (78.7)	93 (84.5)	0.240
Anticoagulant therapy, n (%)	29 (21.3)	17 (15.5)	0.240
Antiplatelet, n (%)	35 (25.7)	27 (24.5)	0.530
Remdesivir, n (%)	74 (54.4)	46 (41.8)	0.049
Convalescent plasma, n (%)	4 (2.9)	3 (2.7)	0.920

**Table 4:** Primary outcomes in the overall population.

**30-Day Mortality**				
	Unadjusted		Adjusted *	
Treatment	OR (95% CI)	*p*	OR (95% CI)	*p*
DEX vs. MP	1.35 (0.71–2.56)	0.351	0.78 (0.35–1.72)	0.544
**s-ICU/ICU Admission**				
	Unadjusted		Adjusted *	
Treatment	OR (95% CI)	*p*	OR (95% CI)	*p*
DEX vs. MP	1.94 (0.81–4.66)	0.136	1.37 (0.54–3.46)	0.503

* Adjusted by age, sex, comorbidities, use of antibiotics, use of Remdesivir, and duration of symptoms before admission. Abbreviations: s-ICU, semi-intensive care unit; ICU, intensive care unit: DEX, dexamethasone; MP, methylprednisolone; OR, odds ratio; CI, confidence interval.

## Supplementary Materials

The following are available online at https://www.mdpi.com/article/10.3390/jcm10245812/s1, Table S1: Symptom presentation at admission, Table S2: Clinical characteristics at admission, Table S3. Primary outcomes in patients with age >80 years (n = 95). Table S4: Primary outcomes in patients with age <80 years (n = 151), Table S5: Rates and type of complications occurring during hospitalization (all data are expressed as n, %).
