# Supplementary material for: Comparison between Dexamethasone and Methylprednisolone Therapy in Patients with COVID-19 Pneumonia Admitted to Non-Intensive Medical Units
Source: J Clin Med. 2021 Dec 12;10(24):5812. doi: 10.3390/jcm10245812 (PMC8703463; doi:10.3390/jcm10245812)
Supplement: Supplementary file 1 [file jcm-10-05812-s001.zip › jcm-1456491-supplementary.pdf]

Table S1: Symptom presentation at admission

Symptom	Methylprednisolone (n=136)	Dexamethasone (n=110)	p
Fever n (%)	117 (86.0)	95 (86.4)	0.940
Dyspnoea n (%)	92 (67.6)	65 (59.1)	0.165
Cough n (%)	69 (50.7)	66 (60.0)	0.147
Myalgia n (%)	8 (5.9)	8 (7.3)	0.660
Headache n (%)	4 (2.9)	3 (2.7)	0.920
Asthenia n (%)	45 (33.1)	50 (45.5)	0.048
Pharyngeal pain n (%)	2 (1.5)	0 (0.0)	0.202
Rhinorrhoea n (%)	4 (2.9)	1 (0.9)	0.261
Anosmia n (%)	2 (1.5)	3 (2.7)	0.487
Dyisgeusia n (%)	3 (2.2)	1 (0.9)	0.424
Gastrointestinal disorders n (%)	18 (13.2)	23 (20.9)	0.108
Syncope n (%)	12 (8.8)	6 (5.5)	0.313

Table S2: Clinical characteristics at admission.

N	Methylprednisolone (n=136)	Dexamethasone (n=110)	p
Heart rate (mean, SD)	85 (17)	87 (17)	0.609
Mean SBP mmHg (mean, SD)	133 (24)	141 (20)	0.013
Mean DBP mmHg (mean, SD)	77 (15)	82 (12)	0.021
Oxygen Saturation % (mean, SD)	93.1 (3.1)	93.0 (4.2)	0.716
Respiratory rate (mean, SD)	23 (6)	22 (5)	0.261
Body temperature °C (mean, DS)	36.8 (1.3)	36.6 (1.0)	0.492
SOFA score (mean, SD)	3.85 (1.4)	4.20 (1.5)	0.330
Padua prediction score (mean, SD)	2.09 (2.2)	1.99 (2.0)	0.683

Abbreviations: SD, standard deviation; SBP systolic blood pressure; DBP diastolic blood pressure

Table S3. Primary outcomes in patients with age ≥ 80 years (n=95).

30 days mortality				
	Unadjusted		Adjusted*	
**Treatment**	**OR (95% CI)**	**p**	**OR (95% CI)**	**p**
**DEX vs. MP**	1.25 (0.54 – 2.92)	0.591	1.46 (0.57 – 3.69)	0.424
**s-ICU/ICU admission**				
	**Unadjusted**		**Adjusted***	
**Treatment**	**OR (95% CI)**	**p**	**OR (95% CI)**	**p**
**DEX vs. MP**	0.54 (0.16 – 1.84)	0.328	0.70 (0.19 – 2.59)	0.598
*Adjusted by sex, comorbidities, use of antibiotics, use of Remdesivir, duration of symptoms before admission				

Abbreviations: s-ICU: semi-intensive care unit; ICU: intensive care unit, DEX Dexamethasone; MP: Methylprednisolone; OR: odds ratio; CI: confidence interval.

Table S4. Primary outcomes in patients with age < 80 years (n=151).

30 days mortality				
	Unadjusted		Adjusted*	
**Treatment**	**OR (95% CI)**	**p**	**OR (95% CI)**	**p**
**DEX vs. MP**	0.64 (0.10 – 3.997)	0.641	1.04 (0.14 – 7.36)	0.969
**s-ICU/ICU admission**				
	Unadjusted		Adjusted*	
**Treatment**	**OR (95% CI)**	**p**	**OR (95% CI)**	**p**
**DEX vs. MP**	0.63 (0.17 – 2.36)	0.502	0.84 (0.20 – 3.58)	0.821

*Adjusted by sex, comorbidities, use of antibiotics, use of Remdesivir, duration of symptoms before admission

Abbreviations: s-ICU: semi-intensive care unit; ICU: intensive care unit, DEX Dexamethasone; MP: Methylprednisolone; OR: odds ratio; CI: confidence interval.

Table S5: Rates and type of complications occurring during hospitalization (all data are expressed as n, %).

	Methylprednisolone (n=136)	Dexamethasone (n=110)	p
All Complications	60 (44.1)	41 (37.3)	0.278
Deep venous thrombosis	0 (0.0)	1 (0.9)	0.265
Pulmonary embolism	8 (5.9)	9 (8.2)	0.480
Haemorrage	1 (0.7)	1 (0.9)	0.880
Atrial Fibrillation (new diagnosis)	8 (5.9)	4 (3.6)	0.416
Heart failure	4 (2.9)	3 (2.7)	0.920
Stroke/TIA	0 (0.0)	1 (0.9)	0.265
Acute renal injury	2 (1.5)	3 (2.7)	0.487
Infections	10 (7.4)	5 (4.5)	0.360
Diabetes (new diagnosis)	3 (2.2)	6 (5.5)	0.177
